# Associated Factors of Atrophic Gastritis Diagnosed by Double-Contrast Upper Gastrointestinal Barium X-Ray Radiography: A Cross-Sectional Study Analyzing 6,901 Healthy Subjects in Japan

**DOI:** 10.1371/journal.pone.0111359

**Published:** 2014-10-24

**Authors:** Nobutake Yamamichi, Chigaya Hirano, Takeshi Shimamoto, Chihiro Minatsuki, Yu Takahashi, Chiemi Nakayama, Rie Matsuda, Mitsuhiro Fujishiro, Maki Konno-Shimizu, Jun Kato, Shinya Kodashima, Satoshi Ono, Keiko Niimi, Satoshi Mochizuki, Yosuke Tsuji, Yoshiki Sakaguchi, Itsuko Asada-Hirayama, Chihiro Takeuchi, Seiichi Yakabi, Hikaru Kakimoto, Ryoichi Wada, Toru Mitsushima, Masao Ichinose, Kazuhiko Koike

**Affiliations:** 1 Department of Gastroenterology, Graduate School of Medicine, The University of Tokyo, Tokyo, Japan; 2 Department of Gastroenterology, Kameda Medical Center Makuhari, Chiba, Japan; 3 Second Department of Internal Medicine, Wakayama Medical University, Wakayama, Japan; Okayama University, Japan

## Abstract

**Background:**

Double-contrast upper gastrointestinal barium X-ray radiography (UGI-XR) is one of the most widely conducted gastric cancer screening methods. It has been executed to find gastric cancer, but has not been usually executed to detect premalignant atrophic mucosa of stomach. To understand the meaning of UGI-XR-based atrophic gastritis, we analyzed its association with several causative factors including *Helicobacter pylori* (HP) infection.

**Methods:**

We evaluated 6,901 healthy adults in Japan. UGI-XR-based atrophic gastritis was diagnosed based on the irregular shape of areae gastricae and its expansion in the stomach.

**Results:**

Of the 6,433 subjects with no history of HP eradication and free from gastric acid suppressants, 1,936 were diagnosed as UGI-XR-based atrophic gastritis (mild: 234, moderate: 822, severe: 880). These were univariately associated with serum HP IgG and serum pepsinogen I/II ratio with statistical significance. The multiple logistic analysis calculating standardized coefficients (β) and odds ratio (OR) demonstrated that serum HP IgG (β = 1.499, OR = 4.48), current smoking (β = 0.526, OR = 1.69), age (β = 0.401, OR = 1.49), low serum pepsinogen I/II ratio (β = 0.339, OR = 1.40), and male gender (β = 0.306, OR = 1.36) showed significant positive association with UGI-XR-based atrophic gastritis whereas drinking and body mass index did not. Among the age/sex/smoking/drinking-matched 227 pairs derived from chronically HP-infected and successfully HP-eradicated subjects, UGI-XR-based atrophic gastritis was detected in 99.1% of the former but in only 59.5% of the latter subjects (*p*<0.0001). Contrastively, UGI-XR-based atrophic gastritis was detected in 13 of 14 HP-positive proton pump inhibitor users (92.9%) and 33 of 34 HP-positive histamine H_2_-receptor antagonist users (97.1%), which are not significantly different from gastric acid suppressant-free subjects.

**Conclusions:**

The presence of UGI-XR-based atrophic gastritis is positively associated with *Helicobacter pylori* infection, current smoking, age, decreased serum pepsinogen I/II ratio, and male gender. Eradication of *Helicobacter pylori* seems to superficially improve UGI-XR-based atrophic gastritis whereas intake of gastric acid suppressants does not.

## Introduction

The incidence and mortality of gastric cancer has gradually fallen in the recent several decades, but it is still the second leading cause of cancer death worldwide [1], [2]. Many gastric cancer screening methods have been developed and executed especially in East Asia, where a high incidence of gastric cancer is observed [3]. Among them, the double-contrast upper gastrointestinal barium X-ray radiography (UGI-XR) is one of the most widely used screening methods for gastric cancer. It has been conducted in Japan since 1960’s as the nationwide mass screening for stomach cancer [3], [4], [5]. Many previous studies suggested that regularly-scheduled UGI-XR may lead to a reduced risk of mortality from gastric cancer [4], [6], [7], [8]. Therefore, UGI-XR is at present the only one method of gastric cancer screening officially authenticated in Japan [9], though other screening methods with endoscopy or serum pepsinogens are gradually spreading [10], [11].

Nowadays, an issue to be solved for UGI-XR-based gastric cancer screening is coming about. UGI-XR has been mainly performed to find gastric cancer and other lesions such as erosion, ulcer, polyp, and so on. But regrettably, atrophic gastritis detected by UGI-XR has not been usually assessed. One of the reasons for that is probably the time when UGI-XR-based gastric cancer screening began: around 1960’s in Japan, the prevalence of *Helicobacter pylori* (*H. pylori*)-induced gastritis was extremely high [3], [5]. In the past several decades, however, the infection rate of *H. pylori* has been decreased worldwide [12], [13], [14], [15]: consequently, clinical significance of evaluating UGI-XR-based atrophic gastritis become relatively higher today. Another more important reason is inadequate validation of the meaning of “atrophic gastritis” diagnosed by UGI-XR. At present, it is well established that chronic *H. pylori* infection mostly causes pathological gastritis with mucosal atrophy and precancerous intestinal metaplasia [16], [17], [18], [19], [20]. However, there is no clear evidence that “UGI-XR-based” atrophic gastritis coincides with *H. pylori*-induced “pathological” gastritis or “endoscopy-based” atrophic gastritis.

Based on these backgrounds, the purpose of this study is evaluating associations of “UGI-XR-based” atrophic gastritis with several causative factors including chronic *H. pylori* infection. Through the large-scale analysis of healthy adults in Japan, we have challenged the unsolved but important problem: the meaning of “atrophic gastritis” diagnosed by UGI-XR. We further expect that our results will improve the efficacy of gastric cancer screening via establishing precise evaluation of premalignant UGI-XR-based atrophic gastritis. Prediction of future cancer risk based on UGI-XR-based atrophic gastritis should increase the value of gastric cancer screening with barium X-ray.

## Materials and Methods

### Study Subjects

The study population was 20,773 subjects who received medical checkup at Kameda Medical Center Makuhari (Chiba-shi, Chiba, Japan) in 2010 and agreed with participating in our study. In cases where health checkup was performed twice in 2010, the former data was used. Criteria for exclusion were insufficient data for analysis or history of gastrectomy. This study was approved by the ethics committee of the University of Tokyo, and written informed consents were obtained from all the study participants according to the Declaration of Helsinki.

### Double-contrast Upper Gastrointestinal Barium X-ray Radiography (UGI-XR)

Five minutes after intramuscular injection of spasmolytic agent (10 mg of scopolamine butylbromide), the subject drank 150 ml of barium (220 w/v %) in one gulp. X-ray images were then taken as follows; 1) double-contrast right anterior oblique view of the upper and lower esophagus in the near-supine standing position, 2) single-contrast frontal view of the stomach in the supine standing position, 3) double-contrast frontal image of the stomach in the supine position, 4) double-contrast right anterior oblique view of the stomach in the near-supine position, 5) double-contrast left anterior oblique view of the stomach in the near-supine position, 6) double-contrast right lateral view of the stomach in the horizontal position, 7) single-contrast frontal view of the stomach in the prone position, 8) double-contrast frontal view of the stomach in the prone position with the head down, 9) double-contrast frontal view of the stomach in the prone standing position, 10) double-contrast left anterior oblique view of the stomach in the prone position with the head down, 11) double-contrast left lateral view of the stomach in the horizontal position, 12) double-contrast left anterior oblique view of the stomach in the near-supine half-standing position (Schatzki’s position), 13) double-contrast left anterior oblique view of the stomach in the near-supine position (“Barium divided” image), and 14) double-contrast right anterior oblique view of the stomach in the near-supine half-standing position.

### Definition of Atrophic Gastritis Based on the Double-contrast Barium X-ray Radiography (UGI-XR-based atrophic gastritis)

**Figure 1:**
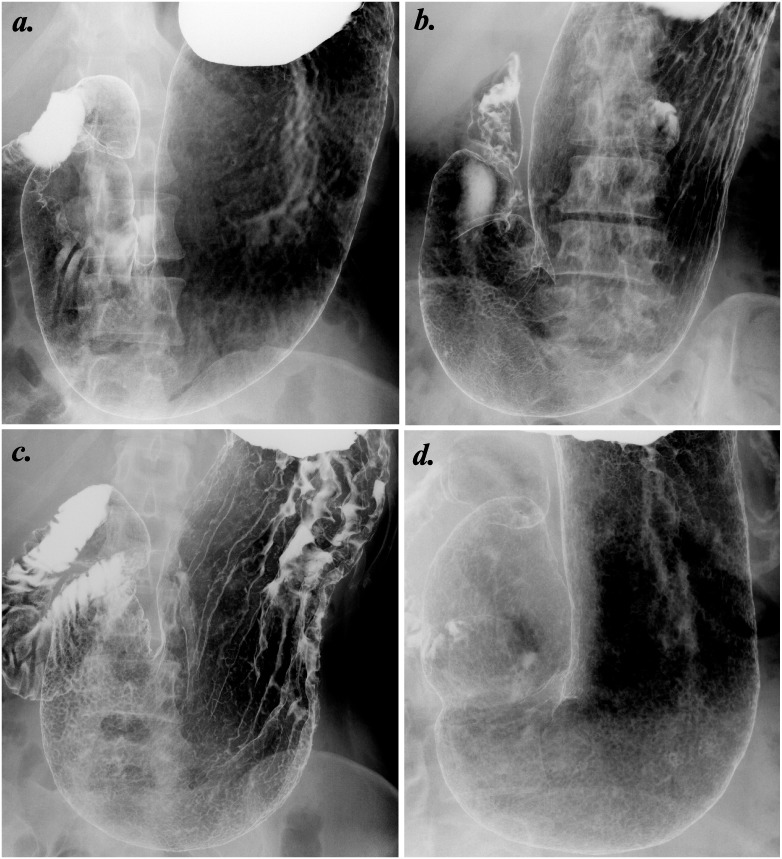
Typical four images of stomach by double-contrast upper gastrointestinal barium X-ray radiography (UGI-XR). (a) Normal stomach with no atrophic change of gastric mucosa. (b) Mild gastritis in which atrophic change mostly exists in gastric antrum and angle, accompanied with slightly enlarged irregular areae gastricae. (c) Moderate gastritis in which atrophic change extends from gastric antrum to body and/or fornix, accompanied with obviously enlarged irregular areae gastricae. (d) Severe gastritis in which atrophic change covers the entire stomach, accompanied with obscured small or even absent areae gastricae.

The characteristics of gastritis in the double-contrast barium X-ray images have been described by a few reports [21], [22]. By referring to them, in our previous report [23], we diagnosed gastritis based on the enlarged areae gastricae and/or hypertrophic gastritis with thickened folds on the greater curvature. It is also well known that atrophic gastritis usually extends from the antrum to body and fornix [19]. Taken these into consideration, we classified the double-contrast barium X-ray images of stomach into four types, on the basis of the irregular shapes of areae gastricae and their expansion as follows;

(A: normal) No atrophic change can be observed in stomach. The areae gastricae cannot be detected or can be recognized as small, round, and regular shapes in all the mucosal surface of stomach (Figure 1a).
(B: mild) The mucosal atrophy is mostly limited to gastric antrum. The enlarged areae gastricae with slight angularity and irregularity are observed in the restricted mucosal surface of stomach (Figure 1b).
(C: moderate) The mucosal atrophy extends from gastric antrum to body (corpus) and/or fornix. The obviously enlarged areae gastricae with considerable angularity and irregularity are observed in most or all mucosal surface of stomach (Figure 1c).
(D: severe) The severe atrophic change entirely covers the mucosal surface of stomach. The small or even absent areae gastricae diffusely extend in stomach, accompanied with irregularly rugged mucosal surface (Figure 1D).

### Disgnosis of Atrophic Gastritis by Endoscopy

Atrophic patterns of gastric mucosa by endoscopy were classified into seven classes according to the Kimura-Takemoto classification [24], [25]: no atrophic change (C0), three closed type atrophy patterns (C1, C2, C3), and three open type atrophy patterns (O1, O2, O3).

### Evaluation of Serum anti-*Helicobacter pylori* IgG, Serum Pepsinogens (PGs), Alcohol Intake, and Smoking

Serum anti-*H. pylori* IgG, pepsinogen I, and pepsinogen II were measured using commercial kits (E-plate “EIKEN” Helicobacter pylori antibody and E-Plate “EIKEN” Pepsinogen I and II, Eiken Chemical Co LTD., Tokyo, Japan) as we had previously reported [26], [27], [28]. According to the manufacture’s instruction, titer of *H. pylori* IgG ≥10 U/ml was considered as *H. pylori*-positive. Recently, it has been suggested that titer of *H. pylori* IgG <10 U/ml should be reconsidered from the standpoint of mucosal atrophic change or gastric cancer risk [29], [30]. Therefore, we further divided “*H. pylori*-negative” subjects into “≥3 and <10 U/ml (gray-zone titer of *H. pylori* IgG)” and “<3 U/ml (absolutely negative for *H. pylori* IgG)”. In accordance with previous reports [28], [31], [32], ratios of serum pepsinogen I and II (pepsinogen I [ng/ml]/pepsinogen II [ng/ml]) were classified into “>3”, “>2 and ≤3”, and “≤2”.

For alcohol intake, the study subjects were scored according to the 5-grade scale (never, seldom, sometimes, often, and always), and further categorized into “rarely drinking” group (never or seldom) and “usually drinking” group (sometimes, often, or always). For smoking, the subjects were classified into three groups: “current smoker” group, “past habitual smoker” group, and “lifelong nonsmoker” group.

### Statistical Analyses

We used JMP 10 software or SAS 9.1.3 (SAS Institute Inc. Cray, NC, USA) for statistical analyses and matching process. In the univariate analysis, associations between the presence of UGI-XR-based atrophic gastritis and seven variables were compared using the χ^2^ test and Cochran-Mantel-Haenszel trend test. In the multivariate analysis, standardized coefficient and odds ratio of each variable were calculated using multiple logistic regression analysis. In the both analyses, *p* values <0.05 were considered as statistically significant.

To estimate the association between UGI-XR-based atrophic gastritis (normal, mild, moderate, and severe) and endoscopy-based atrophic gastritis (C0, C1, C2, C3, O1, O2, and O3 according to Kimura-Takemoto classification) [24], [25], the polychoric correlation coefficient was calculated.

To evaluate the effect of *H. pylori* eradication on UGI-XR-based atrophic gastritis, the matching was performed to control age (±2 years), sex, smoking (current, past habitual, or lifelong non-smoking), and drinking (“rarely” or “usually”) between the successfully *H. pylori*-eradicated subjects (negative for serum *H. pylori* IgG with history of eradication therapy) and the chronically *H. pylori*-infected subjects (positive for serum *H. pylori* IgG with no history of eradication therapy). Using the matched pairs of subjects, we applied Cochran-Mantel-Haenszel trend test, in which *p* value <0.05 was considered as statistically significant.

To evaluate the influence of gastric acid suppressants (proton pump inhibitors (PPI) and histamine H_2_-receptor antagonist (H_2_RA)) upon UGI-XR-based atrophic gastritis, we used Fisher’s exact test in which *p* value <0.05 was considered as statistically significant.

## Results and Discussion

### Characteristics of the Study Subjects

**Figure 2:**
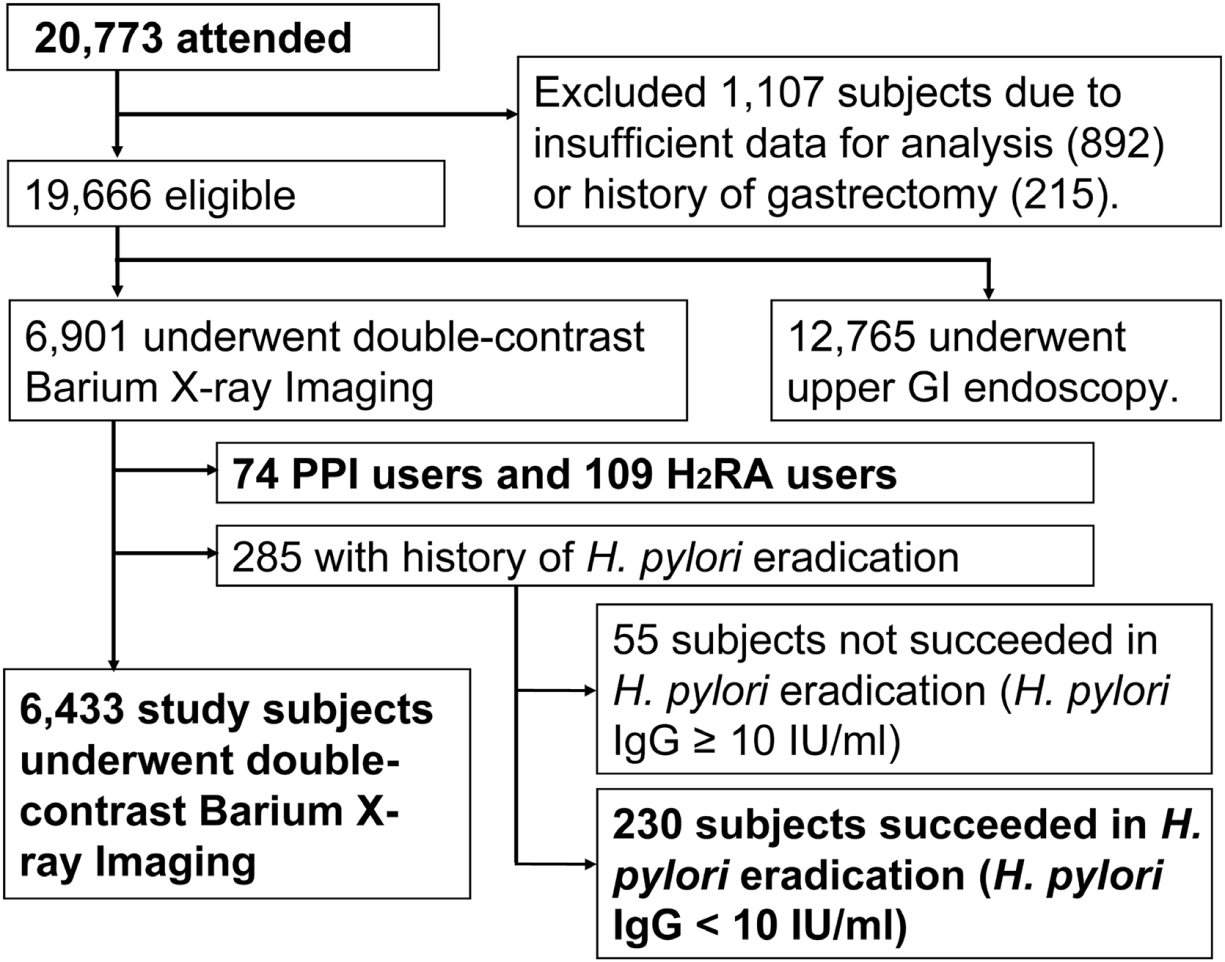
Study flowchart of the present study.

Of the 20,773 subjects who participated in the study (Figure 2), we excluded 1,107 subjects with insufficient data or history of gastrectomy, and also excluded 12,765 subjects who underwent upper gastrointestinal (GI) endoscopy. Of the residual 6,901 subjects, we further excluded 74 PPI users, 109 H_2_RA users, and 285 subjects who had underwent eradication therapy for *H. pylori*. The eligible 6,433 subjects comprised of 3,405 men and 3,028 women (a mean age of 47.4±8.8 years; range 20–83 years) were mainly analyzed in our present study (Figure 2).

Among the 6,433 main subjects for this study, only 1,674 (26.0%) were positive for *H. pylori*, which is consistent with the rapid decrease in prevalence of *H. pylori* infection in Japan [12], [33]. Actually, for the data of healthy adults in our institutes located at Chiba prefecture in Japan, the seropositivity of *H. pylori* infection has markedly reduced from 47.0% (2,695 of 5,732 subjects in 1996–1997 [34]) to 26.0% (1,674 of 6,433 subjects in the present study) in only 14 short years.

The 285 subjects after *H. pylori* eradication therapy comprised of 230 subjects with serum *H. pylori* IgG <10 U/ml (certainly succeeded in *H. pylori* eradication) and 55 subjects with serum *H. pylori* IgG ≥10 U/ml (probably not succeeded in *H. pylori* eradication or on the way of negative conversion of serum *H. pylori* IgG). Aside from the main 6,433 study subjects, we additionally analyzed the above-mentioned 74 PPI users, 109 H_2_RA users, and 230 “successfully *H. pylori*-eradicated” subjects (Figure 2).

### Validation of our defined “UGI-XR-based” Atrophic Gastritis by comparing “Endoscopy-based” Atrophic Gastritis

**Figure 3:**
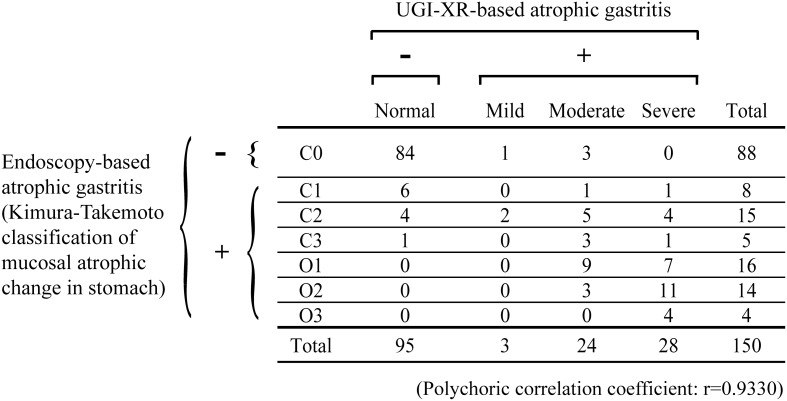
Relationship between the four grades of UGI-XR-based atrophic gastritis and the extent of endoscopy-based atrophic gastritis classified into seven categories according to the Kimura-Takemoto classification (C0 with no atrophic change and C1-O3 with various degrees of endoscopy-based atrophic change of gastric mucosa).

In our previous work [23], 29 (97%) of 30 subjects positive for serum anti-*H. pylori* IgG were diagnosed as gastritis by UGI-XR, which convinced us the sufficient detection of *H. pylori*-induced chronic gastritis by barium X-ray. In the present study, we classified the UGI-XR-based atrophic gastritis into four types as above-mentioned (Figure 1). To validate this classification, the extent of endoscopy-based atrophic gastritis were simultaneously evaluated among the 150 subjects randomly selected (Figure 3).

On the basis of endoscopy-based atrophic gastritis, sensitivity and specificity of UGI-XR-based atrophic gastritis were 82.3% (51/62) and 95.5% (84/88) respectively. In addition, the four-grade categories of UGI-XR-based atrophic gastritis showed significant association with seven-grade classes of endoscopy-based atrophic gastritis (polychoric correlation coefficient: r = 0.9330). Actually, all the subjects (34/34) with severe endoscopy-based atrophic gastritis (namely, open type (O1–O3) atrophy according to Kimura-Takemoto classification [24], [25]) were diagnosed as UGI-XR-based atrophic gastritis (Figure 3). Based on these results, we concluded that UGI-XR-based diagnosis used in this study can certainly reflect the atrophic mucosa of stomach.

### The Four-grade Types of UGI-XR-based Atrophic Gastritis are Significantly Associated with the Titer of Serum *H. pylori* IgG and the Ratio of Serum Pepsinogen I and II

**Figure 4:**
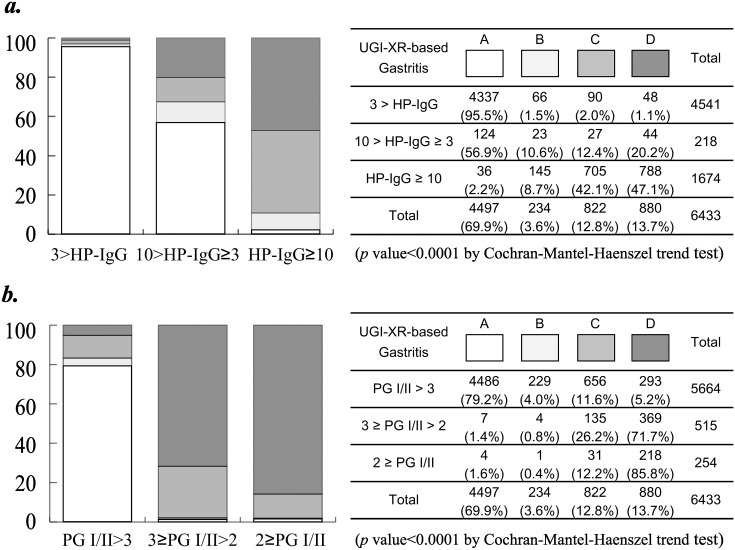
Distribution of our proposed four types of atrophic gastritis by double-contrast upper gastrointestinal barium X-ray radiography (A: normal, B: mild, C: moderate, D: severe) with titer of serum *Helicobacter pylori* IgG (a) or serum pepsinogen I/II ratio (b).

Based on the UGI-XR-based mucosal atrophy of stomach, the total 6,433 subjects with no history of *H. pylori* eradication and free from gastric acid suppressants were classified into four classes (Figure 4): 234 subjects with mild gastritis, 822 subjects with moderate gastritis, 880 subjects with severe gastritis, and residual 4,497 subjects without atrophic gastritis (normal).

We first evaluated associations of the four-grade UGI-XR-based atrophic gastritis with two serum markers: the titer of *H. pylori* IgG and the ratio of pepsinogen I and II reflecting the mucosal atrophy of stomach [32], [35]. As shown in Figure 4a, UGI-XR-based atrophic gastritis significantly extends in proportion to rise in serum *H. pylori* IgG titer (*p*<0.0001). And as also shown in Figure 4b, the grade of UGI-XR-based atrophic gastritis meaningfully advances accompanied with decline in pepsinogen I/II ratio (*p*<0.0001). Though the effects of other causative factors should not be groundlessly underestimated, these results suggest that the four-grade categorization of UGI-XR-based atrophic gastritis strongly reflects chronic *H. pylori* infection and consequent mucosal atrophy of stomach.

### Associated Background Factors of UGI-XR-based Atrophic Gastritis

The detailed characteristics of the 6,433 study subjects focusing on UGI-XR-based atrophic gastritis and the seven putative background factors are shown in Table 1. The results of univariate analyses concerning the seven factors are also denoted. It is clear that old age, male gender, a high titer of serum *H. pylori* IgG, low ratio of serum pepsinogen I/II, and a habit of smoking show strongly positive association with the presence of UGI-XR-based atrophic gastritis.

**Table 1:** Characteristics of the study subjects from the standpoint of atrophic gastritis diagnosed by double-contrast upper gastrointestinal barium X-ray radiography (UGI-XR-based atrophic gastritis).

Factor	Total 6,433study subjects	1,936 subjectswith UGI-XR-based atrophic gastritis	234 subjects with mildUGI-XR-based atrophicgastritis	822 subjects withmoderateUGI-XR-basedatrophic gastritis	880 subjects withsevere UGI-XR-basedatrophic gastritis	Residual 4,497 subjectswithout UGI-XR-basedatrophic gastritis(normal)	*p* value
Age	47.7±8.8 y.o.	51.1±8.9 y.o.	48.4±8.4 y.o.	49.8±8.8 y.o.	53.0±8.7 y.o.	45.8±8.3 y.o.	<.0001*
<30	53 (0.8%)	5 (0.3%)	0 (0.0%)	3 (0.4%)	2 (0.2%)	48 (1.1%)	
≥30 and <40	1,274 (19.8%)	212 (11.0%)	40 (17.1%)	107 (13.0%)	65 (7.4%)	1,062 (23.6%)	
≥40 and <50	2,579 (40.1%)	600 (31.0%)	94 (40.2%)	286 (34.8%)	220 (25.0%)	1,979 (44.0%)	
≥50 and <60	1,909 (29.7%)	774 (40.0%)	76 (32.5%)	304 (37.0%)	394 (44.8%)	1,135 (25.2%)	
≥60 and <70	558 (8.7%)	308 (15.9%)	22 (9.4%)	111 (13.5%)	175 (19.9%)	250 (5.6%)	
≥70	60 (0.9%)	37 (1.9%)	2 (0.9%)	11 (1.3%)	24 (2.3%)	23 (0.5%)	
Sex							<.0001*
female	3,028 (47.1%)	828 (42.8%)	77 (32.9%)	366 (44.5%)	385 (43.8%)	2,200 (48.9%)	
male	3,405 (52.9%)	1,108 (57.2%)	157 (67.1%)	456 (55.5%)	495 (56.3%)	2,297 (51.1%)	
BMI	22.8±3.4	23.0±3.3	23.3±3.4	22.9±3.3	22.8±3.4	22.8±3.4	0.0079*
<18.5	451 (7.0%)	120 (6.2%)	17 (7.3%)	42 (5.1%)	61 (6.9%)	331 (7.4%)	
≥18.5 and <25	4,508 (70.1%)	1,337 (69.1%)	147 (62.8%)	587 (71.4%)	603 (68.5%)	3,171 (70.5%)	
≥25	1,474 (22.9%)	479 (24.7%)	70 (29.9%)	193 (23.5%)	216 (24.5%)	995 (22.1%)	
*H. pylori* IgG							<.0001*
<3	4,541 (70.6%)	204 (10.5%)	66 (28.2%)	90 (10.9%)	48 (5.5%)	4,337 (96.4%)	
<10 and ≥3	218 (3.4%)	94 (4.9%)	23 (9.8%)	27 (3.3%)	44 (5.0%)	124 (2.8%)	
≥10	1,674 (26.0%)	1,638 (84.6%)	145 (62.0%)	705 (85.8%)	788 (89.5%)	36 (0.8%)	
PG I/II ratio							<.0001*
>3	5,664 (88.0%)	1,178 (60.9%)	229 (97.9%)	656 (79.8%)	293 (33.3%)	4,486 (99.8%)	
≤3 and >2	515 (8.0%)	508 (26.2%)	4 (1.7%)	135 (16.4%)	369 (41.9%)	7 (0.2%)	
≤2	254 (3.9%)	250 (12.9%)	1 (0.4%)	31 (3.8%)	218 (24.8%)	4 (0.1%)	
Smoking							<.0001*
non smoker	3,511 (54.6%)	950 (49.1%)	92 (39.3%)	422 (51.3%)	436 (49.5%)	2,561 (57.0%)	
former smoker	1,622 (25.2%)	548 (28.3%)	73 (31.2%)	197 (24.0%)	278 (31.6%)	1,074 (23.9%)	
current smoker	1,300 (20.2%)	438 (22.6%)	69 (29.5%)	203 (24.7%)	166 (18.9%)	862 (19.2%)	
Alcohol							0.8881
rarely drinking	2,567 (39.9%)	770 (39.8%)	75 (32.1%)	319 (38.8%)	376 (42.7%)	1,797 (40.0%)	
usually drinking	3,866 (60.1%)	1,166 (60.2%)	159 (67.9%)	503 (61.2%)	504 (57.3%)	2,700 (60.0%)	

BMI, body mass index; *H. pylori*, *Helicobacter pylori*; PG, pepsinogen. The levels of significance (*p* value) for analyzing associations between UGI-XR-based atrophic gastritis and the seven causative factors were set at <0.05 (*), which were calculated by χ^2^ test or Cochran-Mantel-Haenszel trend test.

We next executed multivariate analyses with these seven causative factors (Table 2). As was expected, a high titer of serum *H. pylori* IgG is the strongest associated factor for UGI-XR-based atrophic gastritis. Current smoking, old age, low ratio of serum pepsinogen I/II, and male gender also show significant association. In contrast, drinking as well as BMI (body mass index) has no meaningful association with UGI-XR-based atrophic gastritis: this unexpected but clear difference between drinking and smoking should be noted when considering the establishment of atrophic gastritis.

**Table 2:** Multivariate analysis of the 6,433 study subjects evaluating associations of the seven background factors with UGI-XR-based atrophic gastritis (atrophic gastritis diagnosed by double-contrast upper gastrointestinal barium X-ray radiography).

Factor	Standardized coefficients	Odds ratio (95% C.I.)	*p* value
Age	0.401	1.49 (1.31–1.70)	<.0001*
Sex			
female	reference	reference	reference
male	0.306	1.36 (1.16–1.59)	0.0002*
BMI	−0.100	0.90 (0.80–1.03)	0.124
*H. pylori* IgG			
<3	reference	reference	reference
<10 and ≥3	0.479	1.61 (1.52–1.72)	<.0001*
≥10	1.499	4.48 (4.12–4.91)	<.0001*
PG I/II ratio			
>3	reference	reference	reference
≤3 and >2	0.270	1.31 (1.18–1.48)	<.0001*
≤2	0.339	1.40 (1.26–1.59)	<.0001*
Smoking			
non smoker	reference	reference	reference
former smoker	0.137	1.15 (0.98–1.33)	0.0773
current smoker	0.526	1.69 (1.49–1.93)	<.0001*
Alcohol			
rarely drinking	reference	reference	reference
usually drinking	0.051	1.05 (0.92–1.20)	0.449

BMI, body mass index; *H. pylori*, *Helicobacter pylori*; PG, pepsinogen. The level of significance in each factor was set at *p*<0.05 (*).

### Eradication of *Helicobacter pylori* Seems to Superficially Improve UGI-XR-based Atrophic Gastritis

We next tried to evaluate the effect of *H. pylori* eradication upon UGI-XR-based atrophic gastritis. For this purpose, the matching was performed to control age (within±2 years), sex, smoking, and drinking between the 230 subjects succeeded in *H. pylori* eradication (negative for serum *H. pylori* IgG with history of eradication therapy) and the 1,674 subjects with chronic *H. pylori* infection (positive for serum *H. pylori* IgG with no history of eradication therapy).

Between the 227 matched pairs of subjects, prevalences of UGI-XR-based atrophic gastritis were markedly different with statistical significance (Table 3): it was detected in only 59.5% of the *H. pylori*-eradicated subjects but was detected in 99.1% of the chronically *H. pylori*-infected subjects (*p*<0.0001). These suggest that eradication of *H. pylori* diminishes the typical images of UGI-XR-based atrophic gastritis. In other words, chronic infection of *H. pylori* in the past cannot be efficiently detected by UGI-XR, after eradication therapy has been completed.

**Table 3:** Comparison between the matched pairs of 227 subjects with chronic infection of *H. pylori* and after successful eradication of *H. pylori*, focusing on the presence of UGI-XR-based atrophic gastritis (atrophic gastritis diagnosed by double-contrast upper gastrointestinal barium X-ray radiography).

	Presence of UGI-XR-basedatrophic gastritis(mild, moderate, severe)	Absence of UGI-XR-basedatrophic gastritis (normal)	Total
The 227 matchedsubjects aftersuccessful eradicationof *H. pylori*	135 (59.5%)	92 (40.5%)	227 (100%)
The 227 matchedsubjects withchronic *H. pylori* infection	225 (99.1%)	2 (0.9%)	227 (100%)

(*p*<0.0001 by Cochran-Mantel-Haenszel test).

It has been reported that eradication of *H. pylori* can improve gastritis both pathologically [20], [36], [37] and endoscopically [38]. The result of our present study suggests that eradication of *H. pylori* can also relieve UGI-XR-based atrophic gastritis, which is defined by the irregular shapes of areae gastricae and their expansion in the stomach. However, this is not always a preferable result, since the superficial improvement of chronic gastritis does not considerably reduce the risk of gastric tumorigenesis [39], [40], [41], [42]. It can be otherwise considered that UGI-XR cannot adequately distinguish the lifelong *H. pylori*-negative stomach (having a very low risk of gastric cancer [17], [30], [43]) from the *H. pylori*-eradicated stomach keeping a considerable risk for gastric canceration [40]. We are apprehensive that the difficulty in detecting *H. pylori*-eradicated stomach will be a formidable problem for UGI-XR-based gastric cancer screening.

### Intakes of PPI and H_2_RA Mostly do not Affect UGI-XR-based Atrophic Gastritis

We further evaluated the influence of gastric acid suppressants upon UGI-XR-based atrophic gastritis. Among the gastric acid suppressant users positive for serum *H. pylori* IgG (Table 4), UGI-XR-based atrophic gastritis was detected in 13 of 14 PPI users (92.9%) and 33 of 34 H_2_RA users (97.1%). Though the statistical evaluation cannot be accurately calculated due to the small number of subjects, there seems to be no obvious differences compared to the *H. pylori*-positive 1,674 subjects free from gastric acid suppressants. To say the least, our results indicate that intakes of PPI and H_2_RA mostly do not deteriorate the diagnostic quality of UGI-XR-based atrophic gastritis.

**Table 4:** Comparison between the *H. pylori*-positive gastric acid suppressant (PPI or H_2_RA) users and *H. pylori*-positive gastric acid suppressant-free subjects, focusing on the presence of UGI-XR-based gastritis (gastritis diagnosed by double-contrast upper gastrointestinal barium X-ray radiography).

	Presence ofUGI-XR-basedatrophic gastritis(mild, moderate,severe)	Absence ofUGI-XR-basedatrophic gastritis(normal)	Total	*p* value
*H. pylori*-positive and gastric acidsuppressant-free subjects amongthe 6,433 main study subjects	1,638 (97.8%)	36 (2.1%)	1,674 (100%)	reference
*H. pylori*-positive subjects amongthe 74 PPI users	13 (92.9%)	1 (7.1%)	14 (100%)	0.2677
*H. pylori*-positive subjects amongthe 109 H_2_RA users	33 (97.1%)	1 (2.9%)	34 (100%)	0.5286

For the PPI and H_2_RA users each, the level of significance was set at *p*<0.05 (*) by Fisher’s exact test.

## Conclusions

The presence of atrophic gastritis diagnosed by double-contrast upper gastrointestinal barium X-ray radiography (UGI-XR-based atrophic gastritis) is positively associated with *Helicobacter pylori* infection, current smoking, old age, decreased pepsinogen I/II ratio, and male gender. Eradication of *Helicobacter pylori* seems to superficially improve UGI-XR-based gastritis whereas intake of proton pump inhibitors or histamine H_2_-receptor antagonist does not.
